# Non-alcoholic fatty liver disease biomarkers estimate cardiovascular risk based on coronary artery calcium score in type 2 diabetes: a cross-sectional study with two independent cohorts

**DOI:** 10.1186/s12933-024-02161-x

**Published:** 2024-02-13

**Authors:** Damien Denimal, Maharajah Ponnaiah, Anne-Caroline Jeannin, Franck Phan, Agnès Hartemann, Samia Boussouar, Etienne Charpentier, Alban Redheuil, Fabienne Foufelle, Olivier Bourron

**Affiliations:** 1 Center for Translational and Molecular Medicine, INSERM UMR 1231, Dijon, France; 2 Department of Clinical Biochemistry, Dijon Bourgogne University Hospital, Dijon, France; 3 Institute of Cardiometabolism and Nutrition (ICAN), Paris, France; 4 Sorbonne Université, Paris, France; 5 Centre de Recherche des Cordeliers, INSERM UMR_S 1138, Paris, France; 6 Department of Diabetology, Assistance Publique‑Hôpitaux de Paris (APHP), Pitié-Salpêtrière Hospital, 47‑83 Boulevard de l’Hôpital, Paris, France; 7 Laboratoire d’Imagerie Biomédicale INSERM_1146, CNRS_7371 Paris, France; 8 ICT Cardiovascular and Thoracic Imaging Unit, Assistance Publique‑Hôpitaux de Paris (APHP), Pitié Salpêtrière University Hospital, Paris, France

**Keywords:** Type 2 diabetes, FibroMax®, FibroTest®, Coronary artery calcium score

## Abstract

**Background:**

Studies have demonstrated that coronary artery calcification on one hand and non-alcoholic fatty liver disease (NAFLD) on the other hand are strongly associated with cardiovascular events. However, it remains unclear whether NAFLD biomarkers could help estimate cardiovascular risk in individuals with type 2 diabetes (T2D). The primary objective of the present study was to investigate whether the biomarkers of NAFLD included in the FibroMax® panels are associated with the degree of coronary artery calcification in patients with T2D.

**Methods:**

A total of 157 and 460 patients with T2D were included from the DIACART and ACCoDiab cohorts, respectively. The coronary artery calcium score (CACS) was measured in both cohorts using computed tomography. FibroMax® panels (i.e., SteatoTest®, FibroTest®, NashTest®, and ActiTest®) were determined from blood samples as scores and stages in the DIACART cohort and as stages in the ACCoDiab cohort.

**Results:**

CACS significantly increased with the FibroTest® stages in both the DIACART and ACCoDiab cohorts (p-value for trend = 0.0009 and 0.0001, respectively). In DIACART, the FibroTest® score was positively correlated with CACS in univariate analysis (r = 0.293, p = 0.0002) and remained associated with CACS independently of the traditional cardiovascular risk factors included in the SCORE2-Diabetes model [β = 941 ± 425 (estimate ± standard error), p = 0.028]. In the ACCoDiab cohort, the FibroTest® F3-F4 stage was positively correlated with CACS in point-biserial analysis (r_pbi_ = 0.104, p = 0.024) and remained associated with CACS after adjustment for the traditional cardiovascular risk factors included in the SCORE2-Diabetes model (β = 234 ± 97, p = 0.016). Finally, the prediction of CACS was improved by adding FibroTest® to the traditional cardiovascular risk factors included in the SCORE2-Diabetes model (goodness-of-fit of prediction models multiplied by 4.1 and 6.7 in the DIACART and ACCoDiab cohorts, respectively). In contrast, no significant relationship was found between FibroMax® panels other than FibroTest® and CACS in either cohort.

**Conclusions:**

FibroTest® is independently and positively associated with the degree of coronary artery calcification in patients with T2D, suggesting that FibroTest® could be a relevant biomarker of coronary calcification and cardiovascular risk.

*Trial registration*: ClinicalTrials.gov identifiers NCT02431234 and NCT03920683.

## Background

Type 2 diabetes (T2D) confers a two-fold increase in the risk of coronary heart disease [1]. However, the population of patients with T2D is highly heterogeneous in terms of cardiovascular risk. The 10-year cardiovascular risk observed in this population ranges from 6% in the DIAD study, 11.7% in the FIELD study, 14.1% in the CARDS study, 16% in the ADVANCE study, 19% in the ACCORD study and up to 34% in the BARI-2D study [2–7]. Cardiovascular risk assessment remains challenging in individuals with T2D, and current risk scores still underperform to accurately predict cardiovascular events [8]. The SCORE2-Diabetes risk model was recently developed to estimate the 10-year risk of major adverse cardiovascular events in this population, and it may outperform current risk scores although further validations are still needed [9].

The measurement of the coronary artery calcium score (CACS) is recommended by the European Society of Cardiology (ESC) and the European Association for the Study of Diabetes to better assess cardiovascular risk in asymptomatic patients with T2D because CACS is able to predict major adverse cardiovascular events and to improve cardiovascular risk classification in this population [10–14]. However, the routine use of CACS leads to relative radiation exposure, as cautioned by the American Diabetes Association [15]. Identifying easy-to-use biomarkers related to coronary artery calcification and cardiovascular risk in patients with T2D would be particularly interesting to avoid this drawback.

Non-alcoholic fatty liver disease (NAFLD), recently renamed metabolic dysfunction-associated steatotic liver disease (MASLD), is highly prevalent in patients with T2D [16, 17]. Large prospective studies have shown that NAFLD is strongly associated with cardiovascular diseases in the general population and in patients with T2D, independently of traditional cardiovascular risk factors [12, 18–20]. NAFLD, when diagnosed by ultrasound, is predictive of CACS and its progression [21–25]. Beyond liver steatosis, non-alcoholic steatohepatitis (NASH), recently renamed metabolic dysfunction-associated steatohepatitis (MASH), is characterized by liver inflammation associated with varying degrees of fibrosis [17]. FibroMax® corresponds to a combination of four non-invasive panels of biomarkers designed to assess liver steatosis (SteatoTest®), necrosis and inflammation (ActiTest® and NashTest®) and fibrosis (FibroTest®) [26]. While both NAFLD and calcification in the coronary arteries are associated with cardiovascular outcomes, it remains unclear whether NAFLD serum biomarkers may help assess cardiovascular risk in patients with T2D. In the present study, we therefore aimed to explore the link between the NAFLD biomarkers included in the FibroMax® panels and the cardiovascular risk estimated by CACS, in two independent cohorts of patients with T2D and different cardiovascular risk profiles.

## Methods

### Study design

For the present ancillary study, we included patients with T2D enrolled in the DIACART and ACCoDiab studies. DIACART is a prospective monocentric cohort study with a recruitment period from February to October 2014 (ClinicalTrials.gov identifier NCT02431234). The inclusion criteria were as follows: T2D with at least a history of coronary artery disease and/or peripheral arterial occlusive disease and/or women over 60 and men over 50 years. The exclusion criteria were as follows: an estimated glomerular filtration rate (eGFR) < 30 ml/min/1.73m^2^, a history of lower limb angioplasty and/or bypass, type 1 diabetes, immunodeficiency or acute infectious or inflammatory disease at inclusion. Among the 169 patients with T2D recruited in DIACART, 12 were withdrawn for the present study due to lack of FibroMax® results (n = 11) or CACS values (n = 1).

ACCoDiab is a cross-sectional monocentric study with retrospective data collection (ClinicalTrials.gov identifier NCT03920683). The recruitment period extended from January 2014 to May 2017. All patients with type 1 diabetes or T2D with a one-day hospital stay to assess cardiovascular comorbidities were eligible for inclusion unless they had personal history of coronary artery disease. Among the 471 patients with T2D enrolled in ACCoDiab, 11 were excluded for the present study due to a lack of FibroMax® results.

The two studies were approved by local ethics committees. All participants were informed of the study objectives and procedures. They gave written informed consent for participation prior to inclusion.

### Laboratory evaluations and FibroMax® panels

Blood samples were collected after an overnight fast. Routine analytical procedures were performed as previously described [27]. FibroMax® panels (BioPredictive, Paris, France) were used to assess liver steatosis, necrosis, inflammation and fibrosis. FibroTest® included serum alpha-2 macroglobulin, apolipoprotein-A1, haptoglobin, total bilirubin and gamma-glutamyltranspeptidase (GGT), adjusted for age and sex. Fibrosis severity is categorized as no (F0), minimal (F1), moderate (F2) or severe (F3-F4) fibrosis. ActiTest®, which includes the same components than FibroTest® plus alanine-aminotransferase (ALT), assesses inflammatory activity, categorizing it as none (A0), minimal (A1), moderate (A2) or severe (A3). SteatoTest® included the same six components as ActiTest® plus body mass index (BMI), serum cholesterol, triglycerides and fasting glucose, adjusted for age and sex. It is categorized as no (S0), minimal (S1), moderate (S2), and marked or severe (S3) steatosis. Lastly, NashTest® included the same parameters as FibroTest® plus ALT, aspartate-aminotransferase (AST), serum cholesterol, triglycerides, and fasting glycemia, adjusted for age and sex. NashTest® severity is classified as minimal (N1), moderate (N2) or severe NASH (N3). Scores of all FibroMax® panels range from 0.00 to 1.00. The laboratory was blinded to CACS values.

### Imaging for coronary artery calcification

CACS was measured in both cohorts using electrocardiographic-gated multidetector computed tomography (semi-automated software using the calcium score as developed by Agatston) in a cardiothoracic imaging unit by radiologists who were blinded to the results of NAFLD biomarkers. The Agatston score is calculated by adding the value of all calcified coronary lesions (in the left main artery, left anterior descending artery, left circumflex artery and right coronary artery) based on the total area and the maximal density of coronary calcifications. The presence of an individual calcified lesion is based on the computed tomography attenuation threshold of 130 Hounsfield units in contiguous voxels of 1 mm^2^. The following categories were used to describe CACS: 0–10 Agatston units (AU) for absence or minimal calcified plaque, > 10 to 100 AU for mild calcified plaque, > 100 to 400 AU for moderate calcified plaque, and > 400 AU for severe calcified plaque.

### Cardiovascular risk classification

We considered the new SCORE2-Diabetes model, recently developed and validated by the ESC, to accurately estimate cardiovascular risk in patients with T2D [9]. It integrates traditional risk factors such as smoking, systolic blood pressure (SBP), total cholesterol, HDL-cholesterol and eGFR values, as well as factors specific to diabetes patients, namely, age at diagnosis of diabetes and HbA1c. Anthropometric characteristics, including BMI and waist circumference, were also taken into consideration as well-established tools for cardiometabolic risk stratification [28]. Cardiovascular risk categories (very-high, high and moderate) were defined according to the ESC guidelines [10].

### Statistical analysis

Statistical calculations were performed using GraphPad Prism (version 9.5.0) and SPSS (version 25). The skewness of each continuous variable was assessed using Pearson's first skewness coefficient, and values were log10 transformed before any statistical analysis to improve normality when necessary. Regarding the FibroMax® panels, continuous quantitative data (scores) were available in DIACART, but only categorical data (stages) were collected in ACCoDiab (e.g., from F0 to F4 for FibroTest®). Individuals with F3 or F4 stages and A2 or A3 stages were grouped together to reach a significant number of subjects.

Data are shown as mean ± standard deviation (SD) or median [1st-3rd quartiles] for continuous variables and percentage (frequency) for categorical variables. The Student’s t and z tests were used to compare the two cohorts for continuous variables and proportions, respectively. Trends between several categories were assessed with the Jonckheere-Terpstra trend test for continuous variables and with the Cochran-Armitage trend test for proportions.

For the univariate regression analysis, Pearson correlation coefficients (r) were determined for continuous variables. Point-biserial correlation coefficients (r_pbi_) were used to assess the association between continuous variables and categorical variables. A Benjamini–Hochberg procedure was applied to control false positives in multiple testing, using a false discovery rate of 5%. Multivariate analysis was performed by linear regression using the least squares method. The goodness-of-fit of multivariate models with or without FibroTest® were compared using the Akaike information criterion, and the statistical significance was calculated using the extra sum-of-squares F test. A random forest machine learning approach was also performed to predict elevated CACS based on the following traditional cardiovascular risk factors: age, sex (female), smoking, BMI, SBP, nephropathy, retinopathy, low-density lipoprotein (LDL) cholesterol, and diabetes duration. Receiver operating characteristic (ROC) analysis were performed using the robust bootstrapping method with tenfold cross validation.

A two-tailed probability level of 0.05 was considered significant for all statistical analyses.

## Results

### Patient characteristics

Table 1 shows the main clinical and biochemical characteristics of the 157 and 460 patients with T2D included in the present study from the DIACART and ACCoDiab cohorts, respectively. Due to the inclusion criteria, 70% of patients in DIACART and none in ACCoDiab had a history of coronary artery disease. Unsurprisingly, the patients in the DIACART cohort were older, more often male, had more history of tobacco use, higher SBP, lower eGFR, higher CACS, and were more frequently taking preventive treatments for cardiovascular disease than patients enrolled in ACCoDiab (p < 0.0001 for all).

**Table 1:** Patient characteristics

Characteristics	DIACART (n = 157)	ACCoDiab (n = 460)	P-value
General characteristics			
Age, y	66.5 ± 8.4	60.1 ± 9.3	< 0.0001
Sex, % female (n)	22.3 (35)	45.2 (208)	< 0.0001
Diabetes duration, y	16.4 ± 9.5	14.0 ± 9.3	0.009
Smoking (active or past), % (n)	66 (103)	43 (197)	< 0.0001
Retinopathy, % (n)	23 (36)	27 (112)	0.32
BMI, kg/m^2^	28.7 [25.3–32.4]	28.4 [25.3–31.6]	0.68
Waist circumference (cm)	103 ± 14	N.P	N.A
SBP, mmHg	135 ± 17	130 ± 13	< 0.0001
DBP, mmHg	77 ± 10	75 ± 12	0.051
Insulin use, % (n)	50 (78)	43 (198)	0.16
Metformin use, % (n)	78 (122)	84 (387)	0.14
Sulfonylurea use, % (n)	45 (71)	48 (219)	0.73
GLP-1 agonist use, % (n)	7 (11)	13 (61)	0.03
Statin use, % (n)	89 (139)	59 (271)	< 0.0001
Ezetimibe use, % (n)	15 (23)	4 (19)	0.0004
Antiplatelet use, % (n)	82 (128)	21 (96)	< 0.0001
ARB or ACE inhibitors use, % (n)	78 (123)	60 (278)	< 0.0001
Beta-blocker use, % (n)	63 (99)	10 (48)	< 0.0001
Routine biological characteristics			
Fasting glycemia, mmol/L	9.06 ± 3.18	8.27 ± 2.72	0.003
HbA1c, %	7.65 [6.90–8.30]	7.50 [6.80–8.30]	0.28
eGFR, mL/min/1.73m^2^	73 ± 19	88 ± 26	< 0.0001
Albuminuria, mg/g creat	2.3 [0.9–11.7]	1.6 [0.7–4.8]	0.007
TyG index	9.2 ± 0.7	9.1 ± 0.7	0.04
Triglyceridemia, mmol/L	1.66 [1.13–2.41]	1.38 [1.01–2.05]	0.004
Total cholesterolemia, mmol/L	3.92 [3.37–4.41]	4.40 [3.80–5.07]	< 0.0001
LDL-cholesterolemia, mmol/L	1.98 [1.60–2.42]	2.38 [1.87–2.92]	< 0.0001
HDL-cholesterolemia, mmol/L	1.06 [0.88–1.27]	1.16 [0.96–1.45]	0.001
AST, IU/L	25 [22–31]	25 [21–30]	0.84
ALT, IU/L	25 [18–32]	24 [19–33]	0.99
GGT, IU/L	33 [24–50]	31 [22–49]	0.54
CRP, mg/L	1.24 [0.66–3.03]	1.74 [0.98–3.48]	0.039
Coronary artery calcification			
CACS	1035 ± 1039	30 [0–211]	< 0.0001
0–10, % (n)	9.6 (15)	42.0 (193)	< 0.0001
> 10—100, % (n)	8.3 (13)	22.4 (103)	< 0.0001
> 100—400, % (n)	20.4 (32)	18.9 (87)	0.75
> 400, % (n)	61.8 (97)	16.7 (77)	< 0.0001
FibroMax® panels			
FibroTest® score	0.372 ± 0.212	N.P	N.A
F0, % (n)	40.8 (64)	64.3 (296)	< 0.0001
F1, % (n)	26.8 (42)	20.0 (92)	0.12
F2, % (n)	14.0 (22)	6.1 (28)	0.012
F3-F4, % (n)	18.5 (29)	9.5 (44)	0.014
SteatoTest® score	0.539 ± 0.208	N.P	N.A
S0, % (n)	25.2 (39)	25.0 (109)	1.00
S1, % (n)	28.4 (44)	29.1 (127)	0.99
S2, % (n)	18.7 (29)	19.3 (84)	0.99
S3, % (n)	27.7 (43)	26.6 (116)	0.95
NashTest® score	0.50 [0.25–0.50]	N.P	N.A
N0, % (n)	32.7 (51)	32.5 (141)	0.68
N1, % (n)	51.9 (81)	50.5 (219)	0.44
N2, % (n)	15.4 (24)	17.1 (74)	0.96
ActiTest® score	0.12 [0.08–0.18]	N.P	N.A
A0, % (n)	90.4 (142)	87.8 (403)	0.41
A1, % (n)	5.7 (9)	10.5 (48)	0.07
A2-A3, % (n)	3.8 (6)	1.7 (8)	0.31

Data are presented as the mean ± SD (for normally-distributed continuous variables) or median [1st-3rd quartiles] (for skewed continuous variables) or percentage (n), as appropriate

*ACE* angiotensin-converting enzyme inhibitor, *ALT* alanine aminotransferase, *ARB* angiotensin II receptor blocker, *AST* aspartate aminotransferase, *BMI* body mass index, *CACS* coronary artery calcium score, *CRP* C-reactive protein, *DBP* diastolic blood pressure, *eGFR* estimated glomerular filtration rate, *GGT* gamma glutamyltransferase, *GLP-1* glucagon-like peptide-1, *HDL* high-density lipoprotein, *IU* international unit, *LDL* low-density lipoprotein, *N.A.* not applicable, *N.P.* not performed, *SBP* systolic blood pressure, *TyG index* triglyceride-glucose index

Regarding the FibroMax® panels, the proportion of patients in each stage of the SteatoTest®, NashTest® and ActiTest® was similar in the two cohorts, whereas the proportion of patients with a FibroTest® stage F0 was higher in ACCoDiab than in the DIACART cohort (64.3 vs. 40.8%, p < 0.0001). Furthermore, more than 70% of patients with T2D had a SteatoTest® stage ≥ S1 in both cohorts (74.8 vs. 75.0% in DIACART and ACCoDiab, respectively, p = 0.50).

### FibroMax® panels and CACS

**Fig. 1:**
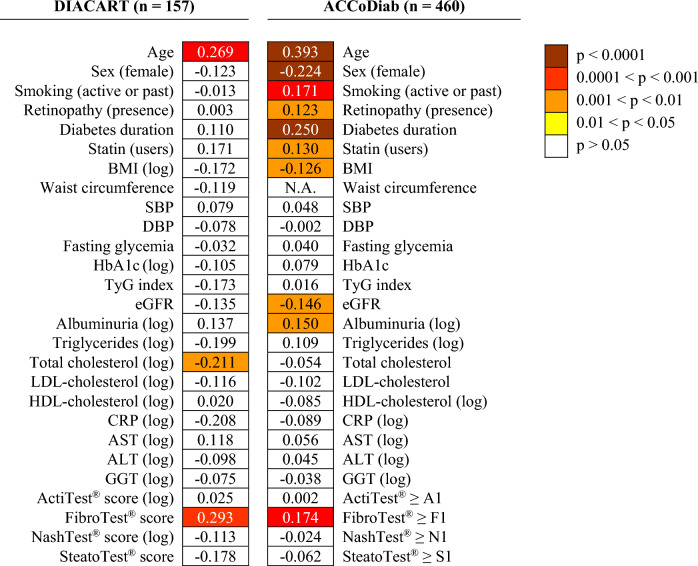
Univariate correlations between coronary artery calcium score and clinical or biological characteristics shown as a heatmap. The figure reports the values of Pearson (r) or point-biserial (r_pbi_) correlation coefficients, as appropriate. Colored boxes indicate instances where the correlation coefficient retained significance following Benjamini–Hochberg correction to control the false discovery rate. CACS values were log10 transformed in the ACCoDiab cohort to improve normality. *ALT* alanine aminotransferase, *AST* aspartate aminotransferase, *BMI* body mass index, *CACS* coronary artery calcium score, *CRP* C-reactive protein, *DBP* diastolic blood pressure, *eGFR* estimated glomerular filtration rate, *GGT* gamma glutamyltransferase, *HDL* high-density lipoprotein, *LDL* low-density lipoprotein, *N.A.* not applicable, *SBP* systolic blood pressure, *TyG index* triglyceride-glucose index

As shown in Fig. 1, FibroTest® was the only FibroMax® panel significantly associated with CACS in univariate analysis in both cohorts [r = 0.293 (p = 0.0002) for the FibroTest® score in DIACART, and r_pbi_ = 0.174 (p = 0.0002) for the FibroTest® ≥ F1 stage in ACCoDiab]. In addition, the FibroTest® score was the only FibroMax® panel score significantly associated with the CACS categories in the DIACART cohort (p-value for trend = 0.002) (Table 2).

**Table 2:** FibroMax® scores according to CACS in the DIACART cohort

FibroMax® scores	Total	CACS				P-value for trend
		0–10	> 10–100	> 100–400	> 400	
n	157	15	13	32	97	
ActiTest® score	0.12 [0.08–0.18]	0.12 [0.08–0.19]	0.10 [0.06–0.22]	0.12 [0.08–0.16]	0.12 [0.07–0.18]	0.98
NashTest® score	0.50 [0.25–0.50]	0.50 [0.25–0.63]	0.50 [0.25–0.50]	0.50 [0.50–0.50]	0.50 [0.25–0.50]	0.24
SteatoTest® score	0.539 ± 0.208	0.584 ± 0.209	0.481 ± 0.183	0.626 ± 0.151	0.512 ± 0.219	0.11
FibroTest® score	0.372 ± 0.212	0.249 ± 0.112	0.275 ± 0.246	0.354 ± 0.201	0.411 ± 0.212	**0.002**

Data are presented as the mean ± standard deviation (for normally-distributed variables) or median [1st–3rd quartiles] (for skewed variables), as appropriate. Bold is used when the p-value < 0.05

*CACS* coronary artery calcium score

A FibroTest® stage ≥ F1 was associated with CACS in both cohorts [r_pbi_ = 0.179 (p = 0.025) and 0.174 (p = 0.0002) in the DIACART and ACCoDiab cohorts, respectively]. This was also the case for a FibroTest® stage ≥ F2 [r_pbi_ = 0.270 (p = 0.0006) and 0.135 (p = 0.004), respectively], which corresponds to the threshold recommended by the European Association for the Study of the Liver to rule out advanced fibrosis in patients with NAFLD [29], and for the FibroTest® F3-F4 stage [r_pbi_ = 0.190 (p = 0.017) and 0.104 (p = 0.024), respectively] (data not shown).

As shown in Table 3, CACS significantly increased along with the FibroTest® stage in both the DIACART and ACCoDiab cohorts (p-value for trend = 0.0009 and 0.0001, respectively). The proportion of patients with a CACS ≤ 10 AU decreased as the FibroTest® stage increased in both cohorts (p-value for trend = 0.011 and 0.007 in the DIACART and ACCoDiab cohorts, respectively). The proportion of patients with a CACS > 400 AU increased as the FibroTest® stage increased in the DIACART and ACCoDiab cohorts (p-value for trend = 0.007 and 0.049, respectively). In the DIACART cohort, no patient with a FibroTest® stage F3-F4 had a CACS between 0 and 10, and almost 90% of these patients had a CACS > 100 AU.

**Table 3:** CACS according to FibroTest® stages

Characteristic	Total	FibroTest® stages				P-value for trend
		F0	F1	F2	F3-F4	
DIACART						
n	157	64	42	22	29	
CACS	1035 ± 1039	812 ± 793	885 ± 937	1424 ± 906	1449 ± 1032	**0.0009**
0–10, % (n)	9.6 (15)	15.6 (10)	9.5 (4)	4.5 (1)	0.0 (0)	**0.011**
> 10–100, % (n)	8.3 (13)	12.5 (8)	4.8 (2)	0.0 (0)	10.3 (3)	0.38
> 100–400, % (n)	20.4 (32)	18.8 (12)	28.6 (12)	22.7 (5)	10.3 (3)	0.43
> 400, % (n)	61.8 (97)	53.1 (34)	57.1 (24)	72.7 (16)	79.3 (23)	**0.007**
ACCoDiab						
n	460	296	92	28	44	
CACS	30 [0–211]	16 [0–173]	61 [0–244]	71 [8–314]	151 [0–600]	**0.0001**
0–10, % (n)	42.0 (193)	47.6 (141)	31.5 (29)	21.4 (7)	36.4 (16)	**0.007**
> 10–100, % (n)	22.6 (103)	22.6 (67)	28.2 (26)	28.6 (8)	6.8 (3)	0.93
> 100–400, % (n)	18.9 (87)	16.9 (50)	22.8 (21)	25.0 (7)	20.5 (9)	0.62
> 400, % (n)	16.5 (77)	12.8 (38)	17.4 (16)	21.4 (6)	36.4 (16)	**0.049**

Data are presented as percentages (n), means ± standard deviations (for normally-distributed variables) or medians [1st—3rd quartiles] (for skewed variables), as appropriate. Bold is used when the p-value < 0.05

*CACS* coronary artery calcium score, *SD* standard deviation

In both cohorts, the odds ratio for having a CACS > 400 AU was significantly higher for patients with a FibroTest® stage ≥ F2 [odds ratio (95% confidence interval) = 2.74 (1.29–5.81), p = 0.009 in DIACART; and odds ratio = 2.66 (1.50–4.74), p = 0.001 in ACCoDiab] and F3-F4 [odds ratio = 2.84 (1.08–7.44), p = 0.034 in DIACART; odds ratio = 3.37 (1.70–6.51), p = 0.001 in ACCoDiab] (data not shown).

### FibroTest® and CACS: multivariate analysis

#### FibroTest® score analysis

 As shown in Table 4, the FibroTest® score was significantly associated with CACS in the DIACART cohort (β = 941 ± 425, p = 0.028), independently of the cardiovascular risk factors included in the SCORE2-Diabetes (i.e. model 1 including age, age at diabetes diagnosis, sex, smoking, SBP, HbA1c, total cholesterol, HDL-cholesterol, and eGFR). The risk equation of the model 1 was as follows: CACS = 941 × FibroTest® score + 36.1 × age (y)—8.7 × age at diabetes diagnosis (y)—230 (if female)—413 (if non-smoker) + 4.2 × SBP -190 × log HbA1c (%)—1884 × log total cholesterol (mmol/L) + 467 × log HDL-cholesterol (mmol/L) + 1.7 × eGFR. The FibroTest® score remained independently associated with CACS in multivariate models including anthropometric characteristics (models 2 and 3). The probability that the multivariate models accurately predicted CACS in DIACART was significantly higher (multiplied by 4.1, 279, and 6.0 for the models 1, 2 and 3, respectively) when the FibroTest® score was added to the models [difference in Akaike information criterion = 2.84 (p = 0.028), 11.3 (p = 0.0003), and 3.57 (p = 0.019) for the models 1, 2

**Table 4:** Multivariate linear regression analysis: variables independently associated with CACS

Variables	Model 1		Model 2		Model 3	
	β coefficient [95% CI]	P-value	β coefficient [95% CI]	P-value	β coefficient [95% CI]	P-value
DIACART (n = 157)						
Age	36 [9.3 to 63]	0.009	N.A	N.A	34 [6.3 to 62]	0.017
FibroTest® score	941 [101 to 1780]	0.028	1424 [662 to 2186]	0.0003	1016 [167 to 1866]	0.019
Total cholesterol (log)	− 1884 [− 3648 to − 119]	0.037	N.A	N.A	− 2092 [− 3897 to − 287]	0.024
Smoking (active)	− 413 [− 896 to 69]	0.093	N.A	N.A	− 387 [− 872 to 98]	0.12
ACCoDiab (n = 460)						
Age	23.4 [15.5 to 31.3]	< 0.0001	N.A	N.A	24.4 [16.3 to 32.4]	< 0.0001
Sex (female)	− 160 [− 279 to − 42]	0.008	N.A	N.A	− 176 [− 296 to − 55]	0.005
FibroTest® F3-F4 stage	234 [43 to 426]	0.016	395 [206—585]	< 0.0001	235 [44 to 427]	0.016
Age at diabetes diagnosis	− 7.7 [− 14.3 to − 1.2]	0.021	N.A	N.A	− 7.5 [− 14.0 to − 0.9]	0.026

**Fig. 2:**
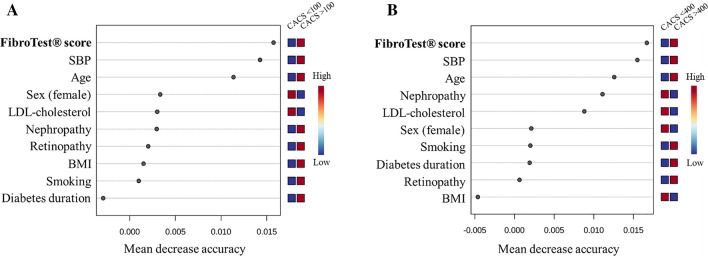
Random forest variable importance plot for predicting CACS > 100 (**A**) and > 400 AU (**B**) in the DIACART cohort. Mean decrease accuracy indicates the importance of each variable in predicting elevated CACS. *BMI* body mass index, *CACS* coronary artery calcium score, *LDL* low-density lipoprotein, *SBP* systolic blood pressure

**Fig. 3:**
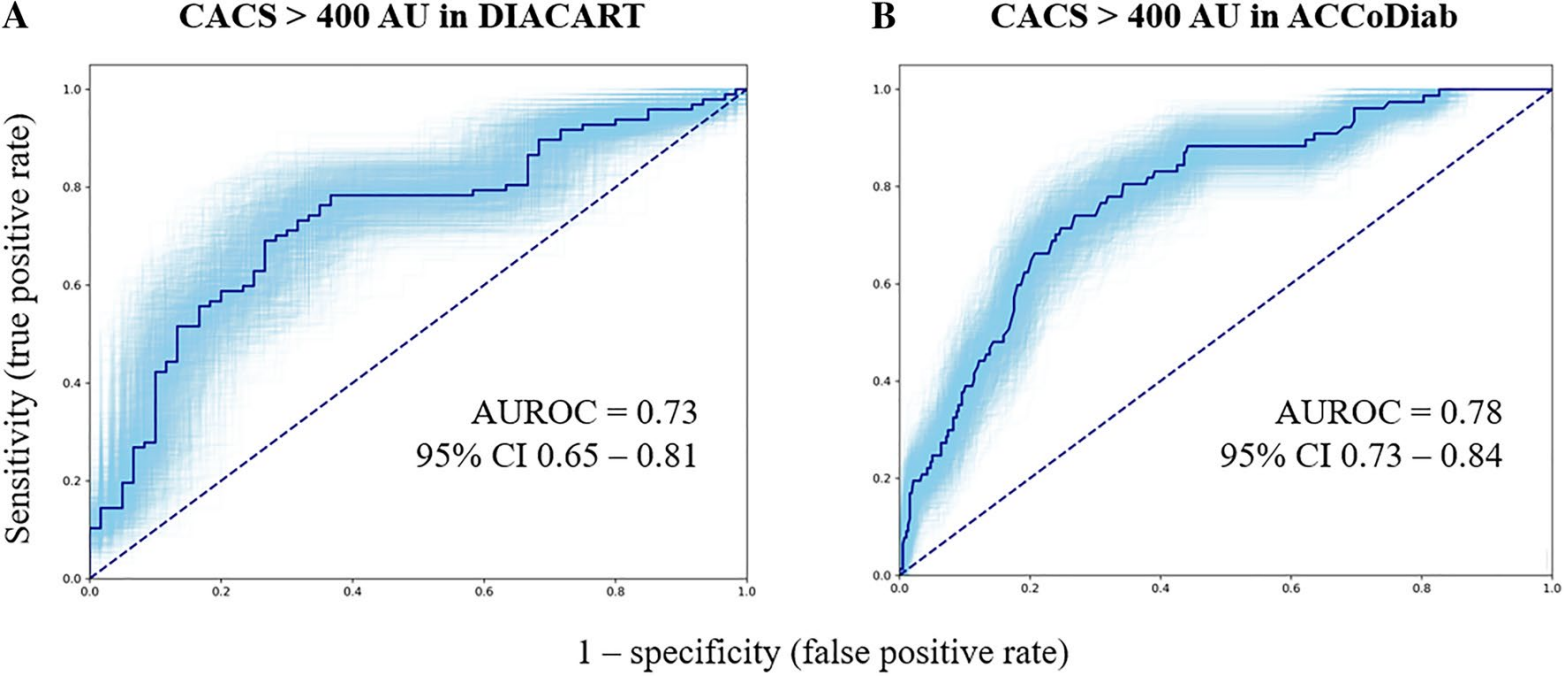
Bootstrapping ROC curves for predicting CACS > 400 AU in the DIACART (**A**) and ACCoDiab (**B**) cohorts. The blue zone represents the 95% confidence interval. *AUROC* area under the receiver operating characteristic curve, *CACS* coronary artery calcium score, *CI* confidence interval

The multivariate model 1 includes FibroTest® and the cardiovascular risk factors included in the SCORE2-Diabetes model [i.e., age, sex (female), age at diabetes diagnosis, smoking (active), systolic blood pressure, total cholesterol, HDL-cholesterol (log), HbA1c (log), and estimated glomerular filtration rate]. The model 2 includes FibroTest® plus anthropometric characteristics (BMI and waist circumference in the DIACART cohort, and BMI alone in patients from the ACCoDiab cohort, where waist circumference was not measured). The model 3 incorporates all variables from the two preceding models. Only explanatory variables with a p-value lower than 0.1 after multivariate analysis are reported in the Table. *CI* confidence intervals, *N.A.* not applicable

and 3, respectively]. As shown in Fig. 2, the FibroTest® score was the most predictive variable for elevated CACS among the traditional cardiovascular risk factors using a random forest machine learning approach. The bootstrapping ROC analysis

for predicting CACS > 400 AU in the DIACART cohort yielded the following equation: -0.631 + (0.075 × age) + (-4.205 × total cholesterol log) + (-0.018 × eGFR) + (0.814 × FibroTest® score) (Fig. 3A). The optimal cutoff was 0.88, and sensitivity, specificity, predictive and negative values were 70, 72, 80, and 60%, respectively.

#### FibroTest® stage analysis

In the DIACART cohort, the FibroTest® ≥ F2 stage was significantly associated with CACS, independently of the cardiovascular risk factors considered in the SCORE2-Diabetes model (β = 375 ± 182, p = 0.041). In the ACCoDiab cohort, the FibroTest® F3-F4 stage was significantly associated with CACS, independently of the cardiovascular risk factors included in the SCORE2-Diabetes model (model 1, β = 234 ± 97, p = 0.016) (Table 4), while the FibroTest® ≥ F1 and ≥ F2 stages were not [β = 19 ± 65 (p = 0.77) and β = 133 ± 81 (p = 0.10), respectively]. The risk equation of the model 1 in ACCoDiab was as follows: CACS = 234 (if F3-F4 stage) + 23.4 × age (y)—7.7 × age at diabetes diagnosis (y)—160 (if female) + 64 (if smoker) + 0.68 × SBP—675 × log HbA1c (%) + 2.6 × total cholesterol (mmol/L)—48 × log HDL-cholesterol (mmol/L) + 1.3 × eGFR. The FibroTest® F3-F4 stage remained independently associated with CACS after adjustment for anthropometric characteristics (models 2 and 3). The probability that the multivariate models were correct for predicting CACS in ACCoDiab was significantly higher (multiplied 6.7, 1484, and 6.8 times) [difference in Akaike information criterion = 3.80 (p = 0.016), 14.6 (p < 0.0001), and 3.83 (p = 0.016) for the models 1, 2 and 3, respectively] when the FibroTest® F3-F4 stage was added to the models. The bootstrapping ROC analysis for predicting CACS > 400 AU in the ACCoDiab cohort yielded the following equation: -5.275 + (0.105 × age) + [-0.834 × 1 (if male) or 2 (if female)] + [0.191 × FibroTest® stage (0 if F0, 1 if F1, 2 if F2, and 3 if F3-F4)] (Fig. 3B). The optimal cutoff was -1.48, and sensitivity, specificity, predictive and negative values were 74, 73, 37, and 93%, respectively.

### Cardiovascular risk classification

The ESC recommends the use of the SCORE2-Diabetes tool to better assess cardiovascular risk in patients with T2D and proposes CACS as a risk modifier in asymptomatic patients with T2D at moderate or high a priori risk [9, 10]. In the ACCoDiab cohort, in which no patient had a history of coronary artery disease, we selected patients at moderate or high a priori risk according to the ESC criteria. The probability that the multivariate model including the cardiovascular risk factors of the SCORE2-Diabetes model was correct for predicting CACS in the 280 patients from ACCoDiab with moderate or high a priori risk significantly increased by 17 times when the FibroTest® F3-F4 stage was added to the model (difference in Akaike information criterion = 5.66, p = 0.006).

## Discussion

The present study is the first to demonstrate a significant relationship between FibroTest® and CACS in patients with T2D. Interestingly, FibroTest® was associated with CACS, independently of the cardiovascular risk factors included in the new SCORE2-Diabetes model, and was even the most predictive variable for elevated CACS using a forest machine learning approach. This association was observed in two independent cohorts, corresponding to more than 600 patients with T2D, with different cardiovascular risk profiles. This allows us to assume that FibroTest® may be an independent marker of cardiovascular risk, regardless of the patient risk profile.

Four studies, mostly in small cohorts, already suggest that liver stiffness or liver fibrosis biomarkers were associated with CACS or its progression in patients with NAFLD [24, 30–32]. However, these studies were not specifically designed to focus on patients with T2D. Our study highlights that, unlike steatosis and NASH, liver fibrosis is strongly associated with coronary calcification in patients with T2D, and the greater the fibrosis severity is, the higher the coronary calcification burden. This is in accordance with the Diabetes Heart Study, which reported no significant association between liver steatosis assessed by computed tomography and CACS in a cohort containing more than 80% of patients with T2D [33]. In addition, the SteatoTest® S3 stage was not predictive of cardiovascular mortality in patients with T2D [34]. The association between NashTest® and CACS had never been explored before our study. We therefore also showed that the assessment of the extent of steatohepatitis is not associated with coronary artery calcification in patients with T2D. One hypothesis to explain why only FibroTest® is associated with CACS could be that liver fibrosis is the final stage of liver disease, reflecting a long evolution of lipotoxicity and inflammation at both hepatic and systemic levels. This hypothesis is supported by the fact that advanced stages of FibroTest® are associated with the highest CACS values. The relationship between advanced fibrosis and elevated CACS in patients with T2D likely involves interrelated pathomechanisms, including inflammation, insulin resistance, and hepatokine dysregulation [35]. Expanded visceral adipose tissue is a major site of low-grade systemic inflammation, release of free fatty acids and dysregulation of adipokine production, that are mechanisms known to promote both NAFLD development and atherosclerosis [36]. In addition, dysregulated secretion of hepatokines in NAFLD play a role in pathways involved in cardiovascular diseases, particularly insulin resistance and inflammation [36]. Especially, fetuin-A has a special significance since it has been shown to inhibit vascular calcification [37]. Although circulating fetuin-A levels are generally elevated in patients with NAFLD, its levels in patients with liver fibrosis remains controversial [38], and a negative correlation has been observed between circulating fetuin-A and the severity of liver fibrosis [39, 40]. Therefore, further studies designed to explore the role of hepatokines in the association between liver fibrosis and CACS would be of particular interest.

Previous studies have suggested that liver fibrosis is linked to cardiovascular events. Accordingly, the FibroTest® F3-F4 stage was the strongest predictor of cardiovascular mortality in patients with biopsy-proven NAFLD after up to 33 years of follow-up [19]. Regarding non-invasive biomarkers of fibrosis, patients with an elevated NAFLD fibrosis score had a 346% increase in cardiovascular diseases in a prospective study including more than 4000 patients with NAFLD (8% had diabetes) [20]. In addition, the FIB-4 score was independently associated with the presence of coronary artery disease in patients with both NAFLD and T2D [41]. Lastly, the FibroTest® ≥ F2 stage was an independent predictor of cardiovascular events in 900 patients with T2D [34]. Unfortunately, thresholds other than F2 have not been considered in this study.

Concerning the cardiovascular risk classification in our study, the prediction of CACS in patients free of coronary disease at moderate or high a priori risk was significantly improved by adding the FibroTest® F3-F4 stage into the model, suggesting a potential role in cardiovascular risk stratification. Nevertheless, prospective studies with the collection of cardiovascular events are needed to determine whether FibroTest® adds to CACS for downward and upward reclassification.

Although our study used validated biomarkers of NAFLD and included a large number of patients with T2D from two different cohorts with different cardiovascular profiles, it has some limitations. First, the lack of continuous values (scores) for FibroMax® in ACCoDiab made it impossible to carry out a strictly similar approach in the two cohorts for some statistical analyses. Second, we used serum biomarkers to indirectly assess NAFLD severity rather than histological characterization of liver biopsies. Nevertheless, the diagnostic performance of FibroTest® is deemed satisfactory when compared to histology in patients with T2D [42, 43], and, therefore, it is one of the non-invasive tests recommended in clinical guidelines to rule out advanced fibrosis in individuals with NAFLD or metabolic risk factors [29, 44]. Thirdly, our study population was not selected on the basis of the presence of liver steatosis assessed by imaging techniques. Therefore, it would be of interest to investigate whether FibroTest® and CACS remain associated in patients with T2D diagnosed with liver steatosis through imaging, which corresponds to the targeted population for the assessment of liver fibrosis [44]. In addition, we could not compare the results obtained with FibroTest® to the non-patented tests FIB-4 or NAFLD fibrosis score since we did not collect platelet counts. Further investigations are necessary to compare FibroTest® to these easy-to-use non-invasive tests for predicting CACS. Interestingly, a recent observation indicates that the FibroTest® outperforms FIB-4 in patients with T2D for diagnosing advanced fibrosis, suggesting that FibroTest® may hold particular significance in the future management of patients with T2D [45]. Lastly, it is important to note that our study had a cross-sectional design, implying an association between FibroTest® and CACS, but not establishing causation.

## Conclusions

We demonstrate that FibroTest® is associated with CACS in patients with T2D, independently of traditional cardiovascular risk factors. Thus, FibroTest®, beyond the assessment of liver fibrosis, could be an interesting predictor of coronary calcification in patients with T2D. Prospective studies with collection of cardiovascular events are required to determine its reliability for downward and upward reclassification of cardiovascular risk in patients with T2D.

## Data Availability

The datasets used and analyzed in the current study are available from the corresponding author upon reasonable request.

## Abbreviations

ACE: Angiotensin-converting enzyme inhibitor
ALT: Alanine aminotransferase
ARB: Angiotensin II receptor blocker
AST: Aspartate aminotransferase
AU: Agatston unit
BMI: Body mass index
CACS: Coronary artery calcium score
CRP: C-reactive protein
DBP: Diastolic blood pressure
eGFR: Estimated glomerular filtration rate
ESC: European Society of Cardiology
GGT: Gamma glutamyltransferase
GLP-1: Glucagon-like peptide-1
HbA1c: Hemoglobin A1c
HDL: High-density lipoprotein
LDL: Low-density lipoprotein
MASH: Metabolic dysfunction-associated steatohepatitis
MASLD: Metabolic dysfunction-associated steatotic liver disease
N.A.: Not applicable
NAFLD: Non-alcoholic fatty liver disease
NASH: Non-alcoholic steatohepatitis
N.P.: Not performed
SBP: Systolic blood pressure
SD: Standard deviation
T2D: Type 2 diabetes
TyG: Triglyceride-glucose
